# Integrated assembly and motion planning using regrasp graphs

**DOI:** 10.1186/s40638-016-0050-2

**Published:** 2016-11-08

**Authors:** Weiwei Wan, Kensuke Harada

**Affiliations:** 1 Intelligent System Research Institute, Artificial Intelligence Research Center, National Institute of AIST, Tsukuba, Japan; 2 Graduate School of Engineering Science, Osaka University, Osaka, Japan

**Keywords:** Assembly planning, Grasp and regrasp, Motion planning

## Abstract

This paper presents an integrated assembly and motion planning system to recursively find the assembly sequence and motions to assemble two objects with the help of a horizontal surface as the supporting fixture. The system is implemented in both assembly level and motion level. In the assembly level, the system checks all combinations of the assembly sequences and gets a set of candidates. Then, for each candidate assembly sequence, the system incrementally builds regrasp graphs and performs recursive search to find a pick-and-place motion in the motion level to manipulate the base object as well as to assemble the other object to the base. The system integrates the candidate assembly sequences computed in the assembly level incrementally and recursively with graph searching and motion planning in the motion level and plans the assembly sequences and motions integratedly for assembly tasks. Both simulation and real-world experiments are performed to demonstrate the efficacy of the integrated planning system.

## Background

### Introduction

 This paper studies the integrated assembly and manipulation planning using regrasp graphs [1] and a horizontal surface [2]. Given two parts and their relative positions in an assembled structure, our integrated planning system decides (1) which object is used as the base, (2) how to place the base, and (3) how to assemble the second part to the base. The results are the integration of assembly sequences and robot motions.

**Fig. 1:**
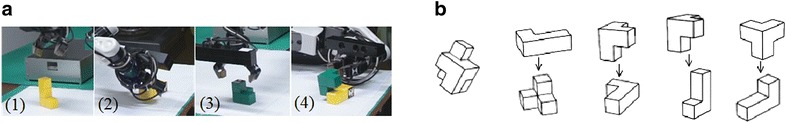
The assembly sequences and motions in state-of-the-art robotic assembly system are manually programmed by technicians using teach pads: Human technicians figure out the assembly plan and program the robot to pick up and place down the base, and assemble the second object to the base (**a**). There are several candidate assembly sequences shown in (**b**), but the technicians manually select one of them using their experience. This paper proposes a novel system to do this automatically using integrated assembly and manipulation planning. **a** The snapshots of assembling two objects. In subfigures (1–2), the robot picks up the *yellow* object and places down it on the table as the base. In subfigures (3–4), the robot picks up the *green* object and assembles it to the base. **b** The possible assembly sequences. The left plot shows the goal. The other plots show different ways of selecting the base and different ways of attaching the second object

In state-of-the-art robotic assembly systems, the assembly sequences and robot motions are pre-defined manually, which significantly impairs the automation of next-generation manufacturing. Take Fig. 1a for example. To use robots to assemble two objects, the traditional way is that technicians figure out the assembly plan and program the robots using teach pads: They make the robot to pick up an object as the base, place it down on a fixture using some pre-defined position and orientation, pick up the second object, and assemble the second object to the base following some given geometric relationship. The assembly sequence is decided by the intelligence of the technicians: There are several possible solutions shown in Fig. 1b, but the technicians manually select one of them for the robot using their experience.

In this paper, we automate the manual process performed by the technicians. We propose an integrated assembly and motion planning system which is done in both assembly level and motion level. In the assembly level, the system checks all combinations of the assembly sequences and gets a set of candidates. In the motion level, the system performs motion planning recursively for each candidate assembly sequence and finds the motions to manipulate the base object as well as to assemble the other object to the base.

Lots of studies have been devoted to individual problems like assembly planning [3], picking-up and placing-down objects [4, 5], regrasping [6–8], as well as motion planning [9]. But to our best knowledge, few studied the integration, which requires considering not only the assembly orders and assembly directions, but also the accessible grasps of each objects and the goal pose of the assembled structure. The later part is an important problem which was never discussed by the assembly planning, grasp planning, and motion planning literature. To this extent, our paper is the initial work that does integrated assembly and motion planning. Specifically, we develop an integrated planning system which considers: (1) Which object should be treated as the base and be manipulated first, (2) which position and orientation should the base be, and (3) how to assemble the second object to the base. Our system plans assembly sequences and motions automatically and can be used to replace some manual work of system integrators. The efficacy of our system is demonstrated using both simulations and real-world executions in the experimental section. The work is expected to be a precedent planner for object assembly using force sensors [10, 11].

### Related work

In respective fields, assembly planning and motion planning are well studied. In assembly planning, early work like [12] and [3] were devoted to symbolic planning. Mello [12] presented the AND/OR graph approach to analyze assembly structures. Wilson and Latombe [3] presented the seminal work that uses non-directional blocking graph (NDBG) to generate assembly sequences. Bozma and Koditschek [13] presented a sphere assembly method which is essentially a path planning problem. More recent work like [14] and [15] concentrate on searching the feasible grasp and manipulation motions with respect to a pre-defined geometric relationships. In particular, [15] does assembly planning from the view point of multiple robot cooperation. Instead of assembly sequences of the objects, [15] plans the assembly sequences of multiple robots. The poses and sequences of objects in the work are pre-defined. Other work like [16] converts assembly planning to a semi-automatic process and learns the assembly sequence from human beings. The adopted method avoids the constraints from the robot bodies and the other parts of the assembled structure, instead of using them. Comparing with our work, it did not integrate the assembly and motion. In motion planning, [17, 18] are the leading study that compared the workspace and joint space approaches. Kavraki et al. [19], Simeon et al. [20] and Lavalle and Kuffner [9] presented the probabilistic approaches to find collision-free motion in the joint space. Vahrenkamp et al. [21], Berenson et al. [22], Phillips and Likhachev [23] represent the more recent studies that use historic data to improve system performance.

For the integration, although there are few studies about integrated assembly and motion planning, there are lots of contemporary research focused on integrated task and motion planning. [24–31] are examples where the planning is done in both task level and motion level. In the task level, these planners employ meta-primitives to divide and conquer tasks. In the motion level, these planners plan motions to implement the primitives generated in the task level. The task-level planning is usually done incrementally and recursively along with the motion-level planning. For example, [28] uses geometric backtracking in task level to decompose and plan the motion in the motion level. Krontiris and Bekris [30] use randomly sampled subgoals as a guide to help motion planners to perform cylinder rearrangements. Dantam et al. [31] present an incremental solution for stacking and rearranging tasks which are effective to exploded combinatorics. The incremental subtasks are alternatives to subgoals in [30] and [32]. Integrate task and motion planning share the same principle with our work, except that we solve the specific task of assembly, and our constraints are from assembly sequences and 6D configurations. These constraints are more complex than picking and moving and require robot to do regrasp [6]. In the assembly level, we check all combinations of the assembly and get a set of candidate assembly sequences. For each sequence, we perform motion planning in the motion level to manipulate the base object and to assemble the other object to the base. The planning in the assembly level is integrated with the planning in the motion level and is done incrementally and recursively by searching regrasp graphs and invoking motion planning algorithms. If the motion planning algorithms cannot find a path in the motion level, we roll back to the assembly level and try another candidate assembly sequence or try a different base until a solution is found or failure is reported.

## Methods

### Overview of the Integrated System

**Fig. 2:**
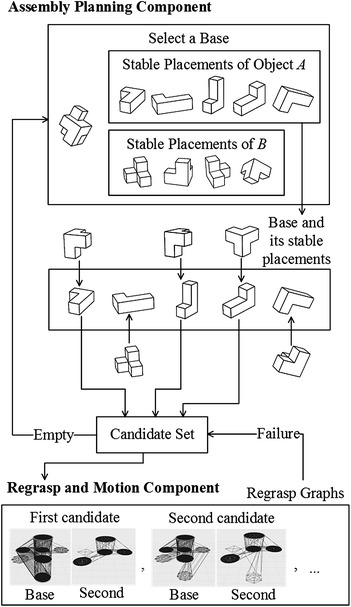
Overview of the integrated assembly and motion planning system

In our work, the integrated assembly and motion planning problem are solved incrementally and recursively by searching regrasp graphs and invoking motion planning algorithms. The overview of the integrated system is shown in Fig. 2. In the first component shown in the upper part of the figure, the system selects a base, computes its placements on the table, and checks the possible sequences of assembling the second object to the placements of the base. The output of the first component is a set of candidate assembly sequences. In the second component shown in the lower part, the system checks each assembly sequence incrementally using regrasp graphs. Each object has an initial state and a goal state. The system computes all the stable placements of the object, connects them with the initial and goal states, and builds a regrasp graph. It searches the regrasp graph to see whether there is a direct motion or a sequence of regrasp motions that the robot can use to pick up the object from the initial state and place it down to the goal state. The regrasp graph is repeatedly built and searched for each object. If the search fails, the system starts over to choose a different candidate sequence or to select a different base.

The details of the two components will be explained in sections “Overview of the integrated system” and “The assembly planning component.” Before that, we list some essential symbols to facilitate readers.

$${\varvec{p}_X}^s$$
The position of Object *X* on a horizontal surface. We use *A* and *B* to denote the two objects, and consequently, ${\varvec{p}_A}^s$ and ${\varvec{p}_B}^s$ are used to denote the positions of the two objects where letter $\varvec{p}$ indicates “position,” and the letter *s* indicates that the object is on a horizontal surface. The notation could denote the initial placements or the positions of intermediate placements on a horizontal surface, depending on the context.
$${\mathbf {R}_X}^s$$
The orientation of Object *X* on a horizontal surface. The letter $\varvec{p}$ indicates “rotation.” Like ${\varvec{p}_X}^s$, *X* is to be replaced by *A* or *B*. ${\mathbf {R}_X}^s$ could denote the initial orientations or the orientations of intermediate placements on a horizontal surface, depending on the context.
$${\varvec{p}_X}^a$$
The position of Object *X* at its assembled state in the assembled structure’s local coordinate system. The letter *a* indicates that the object is at its assembled state.
$${\mathbf {R}_X}^a$$
The orientation of Object *X* at its assembled state in the assembled structure’s local coordinate system.
$${\varvec{p}_X}^{a(g)}$$
The position of Object *X* at its assembled state in global coordinate system. The letter *g* indicates the value is described in global coordinate system.
$${\mathbf {R}_X}^{a(g)}$$
The orientation of Object *X* at its assembled state in global coordinate system.
$${\mathbf {g}_X}^f$$
The force-closure grasps of Object *X*. The letter *f* indicates the object is in a free area without any obstacles. It is neither in an assembled structure nor laying on any surface. *X*’s position and orientation are ([0,0,0], $\mathsf {diag}(1,1,1)$).
$${\mathbf {g}_X}^{s{\prime}}$$
The force-closure grasps of Object *X* on a horizontal surface. Object *X* is at a placement on the horizontal surface. Its position and orientation are (${\varvec{p}_X}^s$, ${\mathbf {R}_X}^s$). The letter *s* indicates the grasps are associated with an object laying on a horizontal surface. The symbol $f^{\prime}$ indicates the grasps are raw, not necessarily collision-free and IK-feasible (IK = inverse kinematics).
$${\mathbf {g}_X}^s$$
The collision-free and IK-feasible grasps of Object *X* on a horizontal surface. Object *X* is at a placement on the horizontal surface. Its position and orientation are also (${\varvec{p}_X}^s$, ${\mathbf {R}_X}^s$). The collision-free and IK-feasible grasps are named accessible grasps.
$${\mathbf {g}_X}^{a(g)^{\prime}}$$
The force-closure grasps of Object *X* at its assembled state. The grasps are described in global coordinate system. *X*’s position and orientation are (${\varvec{p}_X}^{a(g)}$, ${\mathbf {R}_X}^{a(g)}$). The symbol $^{\prime}$ indicates the grasps are raw, not necessarily collision-free and IK-feasible.
$${\mathbf {g}_X}^{a(g)}$$
The collision-free and IK-feasible grasps of Object *X* at its assembled state. The grasps are described in global coordinate system. *X*’s position and orientation are (${\varvec{p}_X}^{a(g)}$, ${\mathbf {R}_X}^{a(g)}$). The collision-free and IK-feasible grasps are named accessible grasps.

### The assembly planning component

The goal of the assembly planning component is to find a set of candidate assembly sequences. The given input to the assembly planning component includes: (1) the relative poses of the two parts, (2) their geometric models, and (3) the position on the supporting table to do assembly. Besides the given input, we assume that the second object is assembled to the base from inverse normal direction of the horizontal surface.

The input (1) means the values of ${\varvec{p}_B}^a$-${\varvec{p}_A}^a$ and ${\mathbf {R}_B}^a\cdot ({\mathbf {R}_A}^a)^{\prime}$ are known. The input (3) means the position of the assembled base ($x_a$, $y_a$) on the table is known. By setting ${\varvec{p}_A}^a$ as the original point and setting ${\mathbf {R}_A}^a$ as the orientation of the assembled structure’s local coordinate system, the variables ${\varvec{p}_X}^a$ and ${\mathbf {R}_X}^a$ can be computed as follows

1 $$\begin{aligned} {\varvec{p}_B}^a&= {\varvec{p}_B}^a - {\varvec{p}_A}^a, \quad {\mathbf {R}_B}^{a} = {\mathbf {R}_B}^{a} \cdot ({\mathbf {R}_A}^{a})^{\prime} \end{aligned}$$

2 $$\begin{aligned} {\varvec{p}_A}^a&= [0, 0, 0]^{\prime}, \quad {\mathbf {R}_A}^{a} = \mathbf {I} \end{aligned}$$

The assembly planning is done based on the placements of the base object. There are two phases in the assembly planning component. The first phase is done in the assembly level. In the first phase, the assembly planner selects a base object (Object *A* for instance), computes all its stable placements on the table, attaches the second object to the base, and computes the collision-free poses of the second objects. We use a set {($\varvec{p}_{Ap}$, $\mathbf {R}_{Ap}$)} to denote the stable placements. For each ($\varvec{p}_{Ap}$, $\mathbf {R}_{Ap}$), the planner computes an assembly candidate $\mathbf {C}_i$ using:

3 $$\begin{aligned}&\mathbf {C}_i = \{({\varvec{p}_{A}}^{a(g)}, {\mathbf {R}_{A}}^{a(g)}), ({\varvec{p}_{B}}^{a(g)},\quad {\mathbf {R}_{B}}^{a(g)}), \end{aligned}$$

4 $$\begin{aligned}&([{\varvec{p}_{B}}^{a(g)}-k\cdot \varvec{n}_t], \quad {\mathbf {R}_{B}}^{a(g)})\} \end{aligned}$$

where

5 $$\begin{aligned} {\varvec{p}_{A}}^{a(g)}&= [x_a, y_a, \varvec{p}_{Ap}.z+h_t],\quad {\mathbf {R}_{A}}^{a(g)} = \mathbf {R}_{Ap} \end{aligned}$$

6 $$\begin{aligned} {\varvec{p}_{B}}^{a(g)}&= {\varvec{p}_{A}}^{a(g)} + {\varvec{p}_B}^a,\quad {\mathbf {R}_{B}}^{a(g)} = {\mathbf {R}_{A}}^{a(g)}\cdot {\mathbf {R}_B}^{a} \end{aligned}$$

and

7 $$\begin{aligned}&\mathsf {S}(\mathsf {A}({\varvec{p}_{A}}^{a(g)}, {\mathbf {R}_{A}}^{a(g)}),\mathsf {B}({\varvec{p}_{B}}^{a(g)}, {\mathbf {R}_{B}}^{a(g)})~\text {is}~\mathsf {TRUE} \end{aligned}$$

8 $$\begin{aligned}&\mathsf {C}(\mathsf {B}({\varvec{p}_{B}}^{a(g)}, {\mathbf {R}_{B}}^{a(g)}),\text {obstacles})~\text {is}~\mathsf {FALSE} \end{aligned}$$

9 $$\begin{aligned}&\mathsf {C}(\mathsf {V}(\mathsf {B}({\varvec{p}_{B}}^{a(g)}, {\mathbf {R}_{B}}^{a(g)}), k\cdot \varvec{n}_t ),\text {obstacles})~\text {is}~\mathsf {FALSE} \end{aligned}$$

$\mathbf {C}_i$ is a triple where the first two elements indicate the pose of the base object and the pose of the second object assembled to it, respectively. The third element is the retraction of the second object from its assembled state along the normal of the supporting horizontal surface. The second object is ensured to be stable in the structure, not colliding with the environment, and assemblable along inverse the horizontal surface normal using expression (7, 8, 9).

In all, the first phase will output a set of candidate assembly sequences:

10 $$\begin{aligned} \mathbf {C}=\{\mathbf {C}_1, \mathbf {C}_2, \ldots , \mathbf {C}_{n}\} \end{aligned}$$

where *n* is equal or smaller than the number of stable placements of the base, depending on the collisions between the second object and the horizontal surface.

The meaning of the unexplained symbols in these equations is as follows. $h_t$ is the height of the horizontal surface. It is a constant value. $\varvec{n}_t$ is the normal of the horizontal surface. ($x_a$, $y_a$) is the position on the horizontal surface to do assembly. Object *A* is treated as the base object. (${\varvec{p}_{B}}^{a(g)}$, ${\mathbf {R}_{B}}^{a(g)}$) is the configuration of object *B* in its assembled state in the global coordinate system. ($[{\varvec{p}_{B}}^{a(g)}-k\cdot \varvec{n}_t]$, ${\mathbf {R}_{B}}^{a(g)}$) is the configuration of Object *B* after being disassembled along the normal of the horizontal surface with a step length *k*. $\mathsf {S}(\mathsf {A}(\varvec{p}, \mathbf {R}),\mathsf {B}(\varvec{p}, \mathbf {R})~\text {is}~\mathsf {TRUE}$ is a logical expression that ensures the structure composed by the two objects at the given poses is stable ($\mathsf {S}$ indicates stable). $\mathsf {C}(\mathsf {X(\varvec{p}, \mathbf {R})}, \text {obstacles})\text {~is~}\mathsf {FALSE}$ is a logical expression that ensures the Object *X* at position $\varvec{p}$ and orientation $\mathbf {R}$ does not collide with the *obstacles* ($\mathsf {C}$ indicates collision). $\mathsf {C}(\mathsf {V}(\mathsf {X}(\varvec{p}, \mathbf {R}), k\cdot \varvec{n}_t),\text {obstacles})~\text {is}~\mathsf {FALSE}$ ensures the swept volume of Object *X* moving along $\varvec{n}_t$ with a step length *k*.

**Fig. 3:**
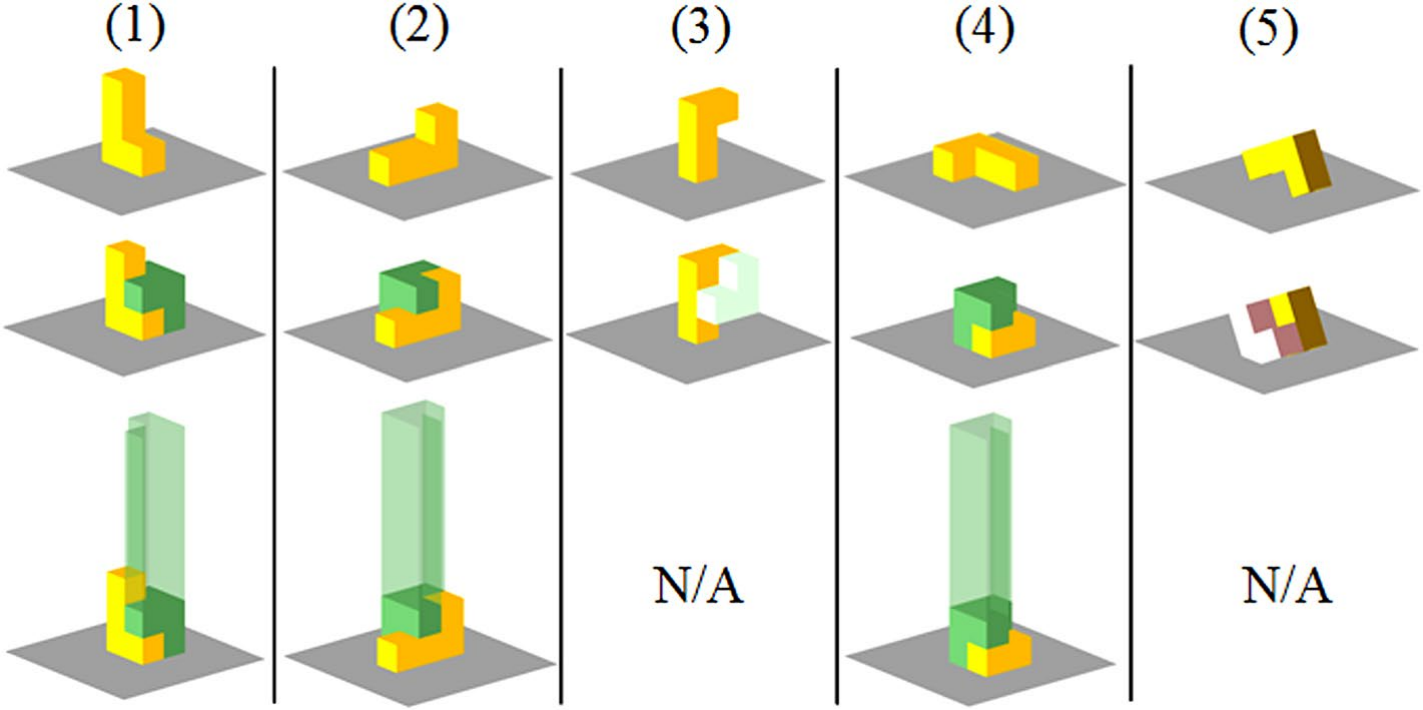
The first phase of the assembly planning component. The *first row* shows the placements of the Object *A* in Fig. 2. The *second row* shows the states after attaching Object *B* to the placements. The *third row* shows the feasibility along the pre-defined assembly directions. Three cases in the *third row* are feasible and outputted as $\mathbf {C}$ by the first phase

Take the two objects in Fig. 2 for example. The Object *A* has five ($\varvec{p}_{Ap}$, $\mathbf {R}_{Ap}$) which are plotted in the first row of Fig. 3 using yellow color. The states after attaching Object *B* to the placements of *A* are shown in the second row of blow each element of the first row. The third case in the second row is not stable. It is detected by expression (7). The unstable object is marked using light green. The fifth case collides with the surface, which is detected by expression (8). The collided object is marked using light pink. The remaining assemblies are stable and collision-free (objects are plotted in dark green). The stable and collision-free candidates are further checked using expression (9) in the third row. All three cases in the third row passed the check and are outputted as $\mathbf {C}$.

The output of the first phase is the output of the assembly planning component and is performed offline. The second phase is part of the assembly planning component, but it does not directly relate to the output. The algorithms in the second phase work in the motion level and are used online with graph building and searching in the regrasp and motion component. In the second phase, the assembly planer interacts with a grasp planner, loops through all elements in ${\mathbf {C}}$, and finds IK-feasible and collision-free grasps that the robot can use move the objects during assembly. The output of the second phase is a subset of ${\mathbf {C}}$ where the objects are at collision-free states and graspable.

Beforehand, the planner sets the two objects at free space and computes the force-closure and collision-free grasps. Each grasp is represented using ${\varvec{g}_X}^f$=$\{\varvec{p}_0, \varvec{p}_1, \mathbf {R}\}$ where $\varvec{p}_0$ and $\varvec{p}_1$ are the contact positions of the finger tips, and ${\mathbf {R}}$ is the orientation of the palm. The whole set is represented by ${\mathbf {g}_X}^f$, which includes many ${\varvec{g}_X}^f$. Namely, ${\mathbf {g}_X}^f$ = $\{{\varvec{g}_X}^f\}$.

Given a triple ${\mathbf {C}}_i$, the IK-feasible and collision-free grasps that the robot can use to assemble it are computed as follows

11 $$\begin{aligned} {\mathbf {g}_X}^{a(g)} = \mathsf {IK}~({\mathbf {g}_X}^{a(g)^{\prime}}) \cap \mathsf {CD}~({\mathbf {g}_X}^{a(g)^{\prime}},~\text {obstacles}) \end{aligned}$$

where

12 $$\begin{aligned} {\mathbf {g}_X}^{a(g)^{\prime}} = {\mathbf {R}_X}^{a(g)}\cdot {\mathbf {g}_X}^f+{\varvec{p}_X}^{a(g)} \end{aligned}$$

If none of the ${\mathbf {g}_X}^{a(g)}$ computed by the three ${\varvec{p}_X}^{a(g)}$ and ${\mathbf {R}_X}^{a(g)}$ in the triple is empty, the triple will be saved as a candidate assembly sequence and will be used to build the regrasp graph and do motion planning.

**Fig. 4:**
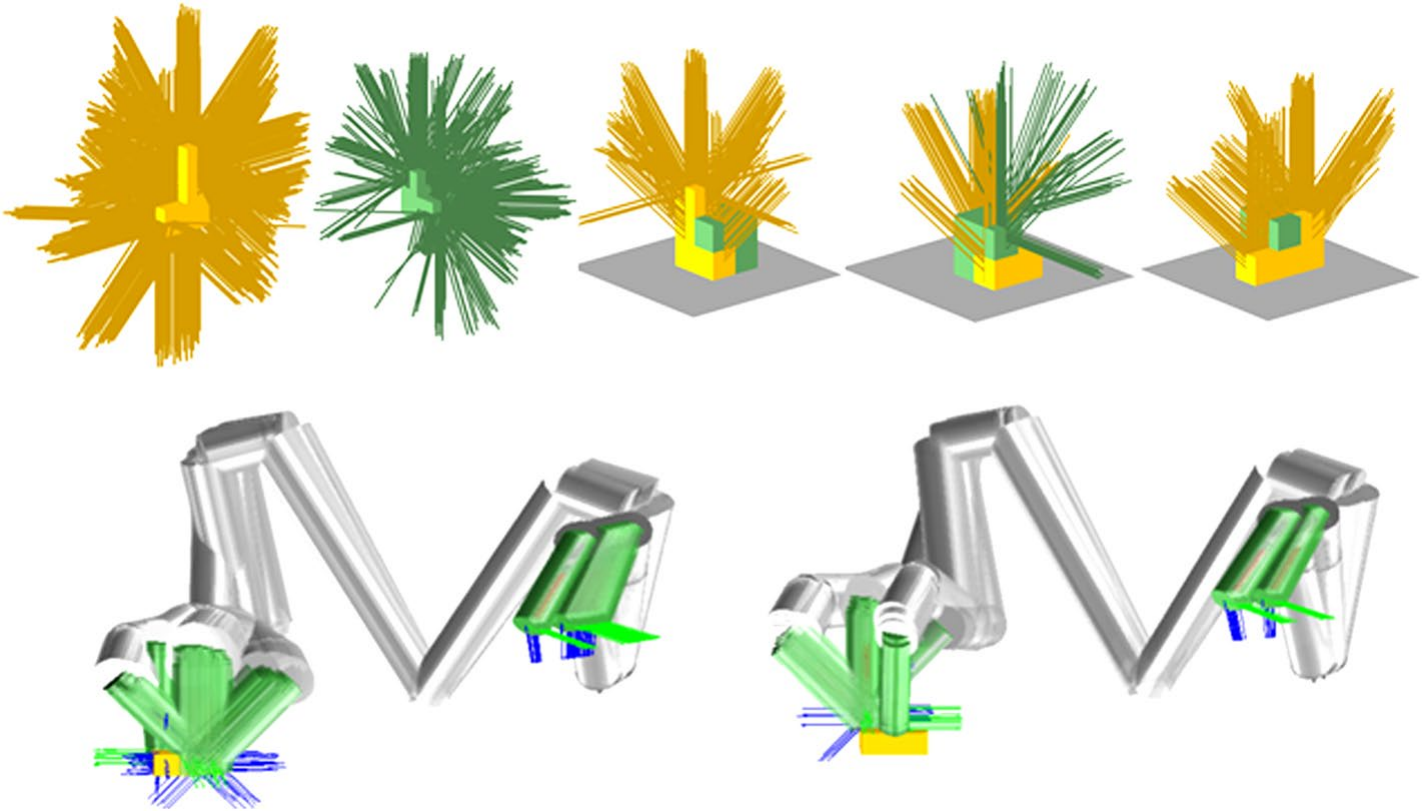
The left two plots of the *first row* show the ${\mathbf {g}_A}^f$ and ${\mathbf {g}_B}^f$ associated with the two objects in Fig. 2 when they are posed at free space. The remaining three plots of the *first row* show the collision-free grasps when the two objects are assembled on a horizontal surface. The two plots in the *second row* show the IK-feasible grasps of the two objects. The kinematic model is from the Kawada Nextage robot

Figure 4 shows some details of the second phase. The left two plots of the first row show the ${\mathbf {g}_A}^f$ and ${\mathbf {g}_B}^f$ associated with the two objects in Fig. 2. The two objects are at free space in these two plots, and no obstacles are nearby. The grasps are plotted as segments to make them easy to recognize. The remaining three plots of the first row show the collision-free grasps when the two objects are assembled on a horizontal surface. The removed grasps either collide with the table surface or collide with the other object in the assembled structure. They are the results of applying $\mathsf {CD}~({\mathbf {g}_X}^{a(g)^{\prime}},~\text {obstacles})$ to $\mathbf {C}_{i(i=1,2,\ldots )}$. The results of further applying $\mathsf {IK}~({\mathbf {g}_X}^{a(g)^{\prime}})$ are shown in the second row of Fig. 4. Robot kinematics are considered in this case (Kawada Nextage^1^).

### The regrasp and motion component

The input to the regrasp and motion component includes: (1) the initial states of the objects, which are obtained using vision systems, and (2) the goal states of the two objects, which are the assembled states and are included in the candidate set ${\mathbf {C}}$ produced by the assembly planning component. The regrasp and motion component incrementally builds regrasp graphs using the initial state, the goal state, and the placements of each candidate in ${\mathbf {C}}$. It recursively searches the regrasp graphs to find sequences of grasps and plans the motion between the grasps using motion planning algorithms.

Given the initial states or the placements of an object on the horizontal surface, say ${\varvec{p}_X}^s$ and ${\mathbf {R}_X}^s$, the IK-feasible and collision-free grasps that the robot can use to pick up the object at these states are computed as follows

13 $$\begin{aligned} {\mathbf {g}_X}^{s} = \mathsf {IK} ({\mathbf {g}_X}^{s^{\prime}}) \cap \mathsf {CD} ({\mathbf {g}_X}^{s^{\prime}},~\text {obstacles}) \end{aligned}$$

where

14 $$\begin{aligned} {\mathbf {g}_X}^{s^{\prime}} = {\mathbf {R}_X}^s\cdot {\mathbf {g}_X}^f+{\varvec{p}_X}^s \end{aligned}$$

Here Eqs. (13, 14) are similar to the ones in (11) and (12) except they are performed on the initial states.

**Fig. 5:**
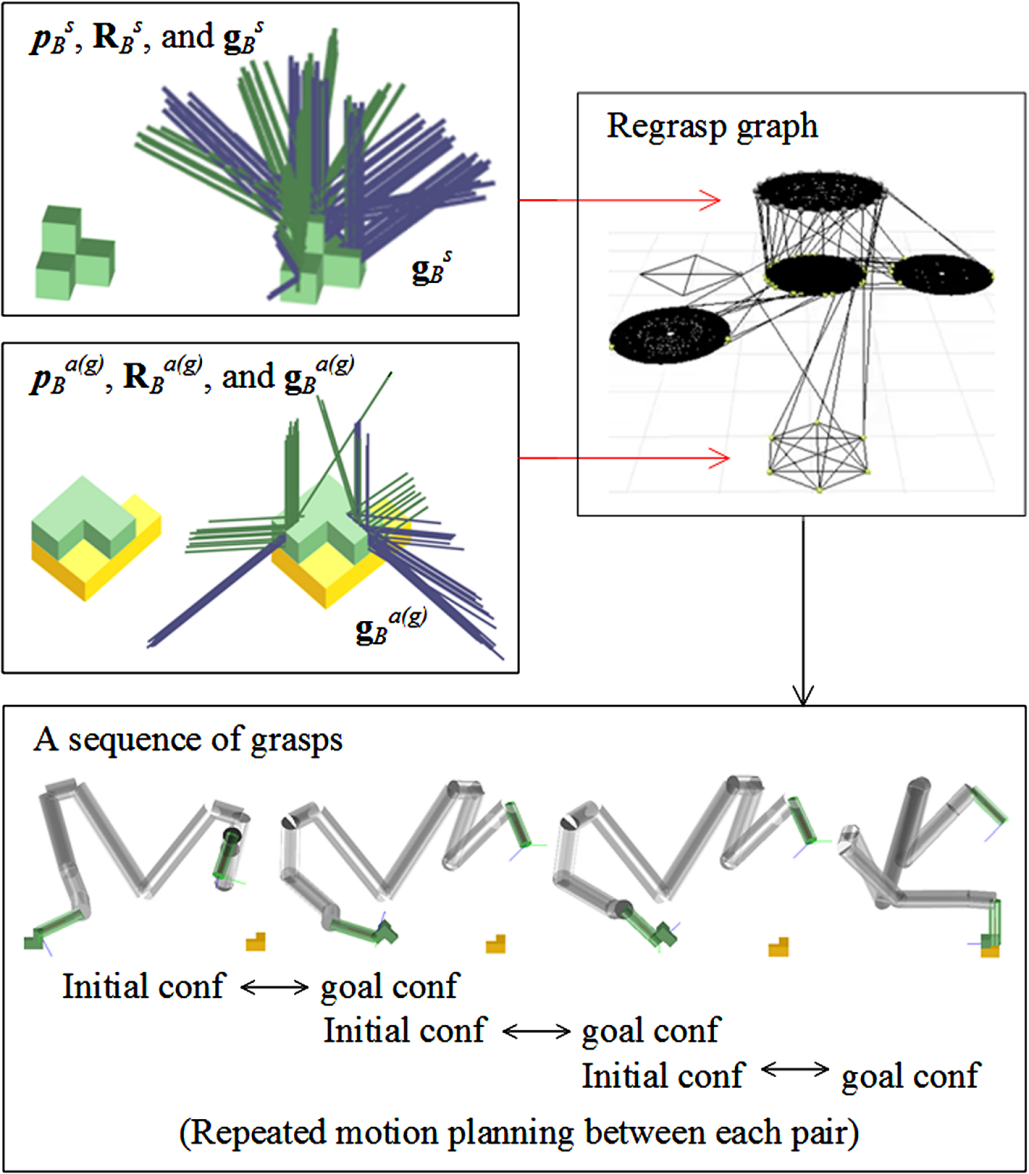
The flow of the regrasp and motion component. Given the initial and goal states of an object (in this case the Object *B* in Fig.2), we compute its accessible (collision-free and IK-feasible) initial and goal grasps, and use these grasps together with the grasps associated with the placements of the object on a horizontal surface to build regrasp graphs. These two steps are shown in the upper part of the flowchart. The *green* segments in the plots denote accessible grasps. The *blue* ones denote the IK-infeasible grasps. We recursively search the regrasp graphs to find a sequence of grasps and do motion planning repeatedly between the grasps to find a solution to the desired task. The robot configurations in the *lower part* of the flowchart show as a sequence of grasps obtained by searching the regrasp graph

Then, the regrasp and motion component builds a graph using the elements in ${\mathbf {g}_X}^{s}$ and ${\mathbf {g}_X}^{a(g)}$. Figure 5 shows the flow using the Object *B* in Fig. 2. The initial pose of the object on the planery surface, namely ${\varvec{p}_B}^s$ and ${\mathbf {R}_B}^s$, is shown in the upper-left plot. When assembled in the structure, its pose ${\varvec{p}_B}^{a(g)}$ and ${\mathbf {R}_B}^{a(g)}$ is shown in the bottom-left plot. The grasps ${\mathbf {g}_X}^{s}$ and ${\mathbf {g}_X}^{a(g)}$ associated with them are shown in the plots besides the two poses. They are rendered using colored segments where green means the grasp is both collision-free and IK-feasible. Blue means the grasp is collision-free, but IK-infeasible. The collided grasps are not shown. A regrasp graph is built based on the common grasps, the initial grasps, goal grasps, and some intermediate placements. The planner recursively searches the regrasp graph, finds a sequence of grasps, and employs transition-RRT to find a motion between the grasps. Note that the planning is done between the grasp associated with the initial states and the third element of the triple in $\mathbf {C}_i$. Directly planning to the grasps associated with the first element is a narrow passage problem [33, 34] and should be avoided.

**Fig. 6:**
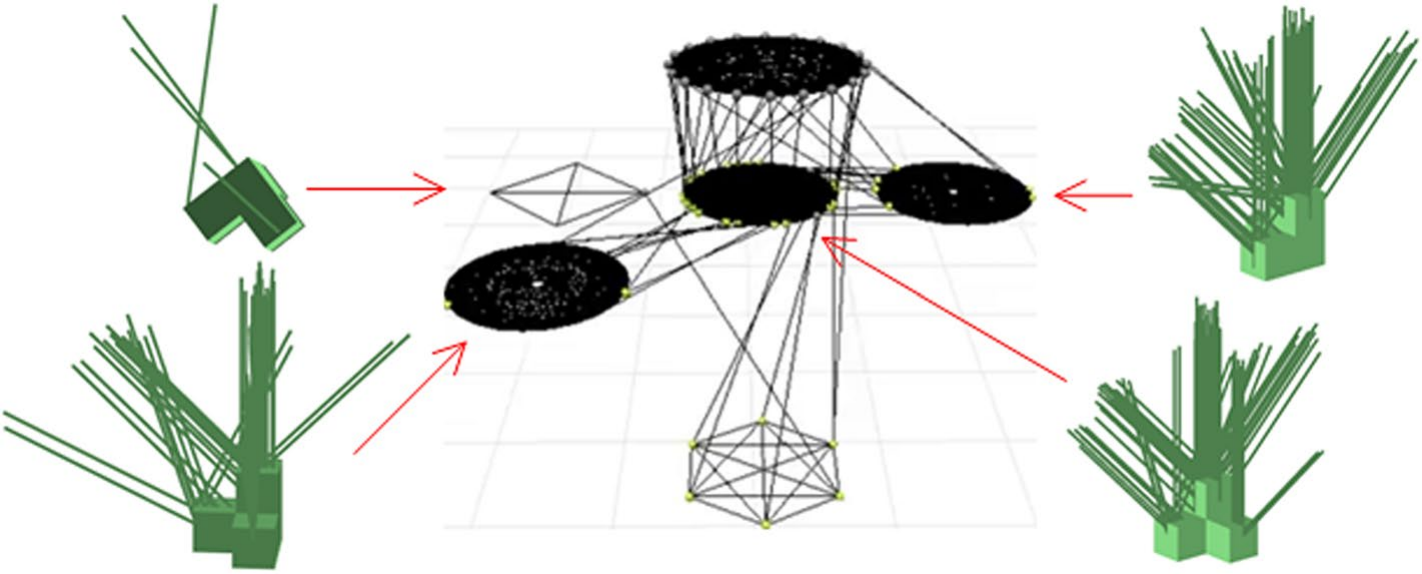
More details about the regrasp graph. The regrasp graph has three layers where the *top layer* and the *bottom layer* have *one circle *, respectively. They encode the initial and goal states and their accessible grasps. The correspondence has been shown by the *red arrows* in Fig. 5. The *middle layer* encodes the placements on horizontal surfaces and their accessible grasps. The four placements and their accessible grasps of Object *B* are plotted in the figure. Their correspondent *circles* in the *middle layer* are denoted using *red arrows*. By searching this regrasp graph, the planner finds a sequence of grasps. The sequence may include some intermediate nodes like the one shown in the *lower part* of Fig. 5. It may also connect directly from the initial state to the goal

More details about the regrasp graph are shown in Fig. 6. It is basically the same as [29], except that we put the initial and goal states and their accessible grasps in the top and bottom layers, respectively. The top layer has only one circle which encodes the initial state of the object and its accessible grasps. The bottom layer also has only one circle which encodes the goal state of the object and its accessible grasps. The red arrows in the upper part of Fig. 5 show the relationship between the initial and goal state, their accessible grasps (collision-free and IK-feasible grasps, plotted as green segments in the figure), and the top and bottom layers of the regrasp graph. The middle layers are composed of several circles where each of them encodes a possible placement on a horizontal surface and its accessible grasps. The arrows in Fig. 6 show the correspondence between the placements of Object *B*, their accessible grasps, and the circles in the middle layer.

Each node on the circles represents a grasp: The ones in circle of the upper layer are from ${\mathbf {g}_X}^s$, and the ones in the circle of the bottom layer are from ${\mathbf {g}_X}^{a(g)}$. The ones from the circles in the middle layer are the grasps associated with the placements. The orientations of the placements are evenly sampled online. Their positions are fixed to a pre-defined position in front of the robot.^2^ If the circles share some grasps (grasps with the same $\varvec{p}_0$, $\varvec{p}_1$, $\mathbf {R}$ values in the object’s local coordinate system), we connect them at the correspondent nodes.

The regrasp and motion component searches the graph to find a sequence of grasps that manipulates the object from its initial state to the goal. For each adjacent pair in the grasp sequence, the regrasp and motion component repeatedly performs motion planning to find feasible motions. If no feasible motion was found, the component roll back to try a different sequence, a different triple in $\mathbf {C}$, or use a different based.

**Fig. 7:**
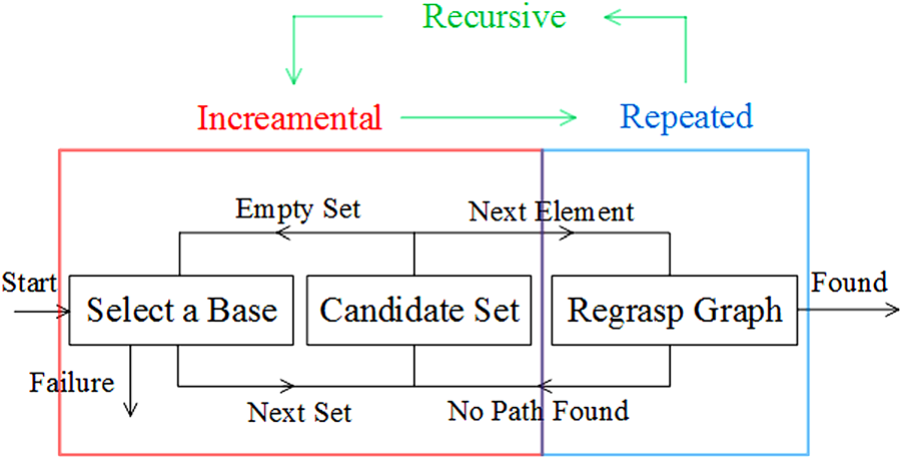
Details of the repeatedly graph building and the recursive search. The algorithm in the component incrementally uses each element in the candidate set to do motion planning. If fails, it tries a different element or a different candidate until all possibility is tested

The regrasp graph is repeatedly built for each object and is recursively searched for motion planning. The details of the repetition and recursion are summarized in Fig. 7. Incrementally for each element in the candidate set, the component builds the regrasp graph and performs motion planning. If the planning succeeds, the component reports “found,” and otherwise, it tries a different element from the candidate set. If all elements in the candidate set are visited, the component tries a different base. Exemplary results will be shown in the experimental section. A disadvantage of the incremental process is the time cost that may approach infinity. To avoid that, we also employed a time limit in implementation. When planning time goes over the limit, our system reports failure.

## Results and discussion

We perform both simulation and real-world experiments to demonstrate the efficacy of the integrated assembly and motion planning system. The computer used to compute the grasps and motions is a Dell T7910 workstation (Processor: Intel Xeon E5-2630 v3 with 8CHT, 20MB Cache, and 2.4GHz Clock, Memory: 32G 2133MHz DDR4). The robot used to perform real-world experiments is Kawada Nextage Open. The relative poses between the two objects are obtained in advance using a teaching system.^3^ The repeatedly invoked motion planning algorithm is transition-RRT [37].

**Fig. 8:**
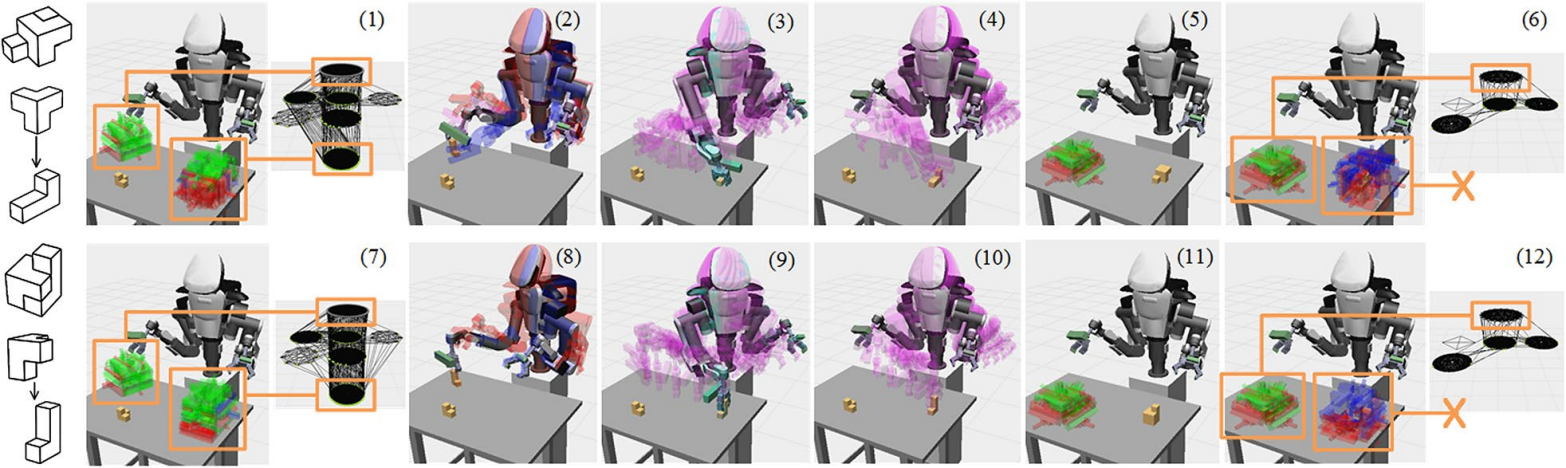
Two failure cases during the repeated graph building and recursive searching. These two cases use the *first* and *second elements* in the candidate assembly sequence of the two objects shown in Fig. 2 to build the regrasp graphs and do motion planning. Both of them failed since there are no accessible grasps to place down the second object to its goal states. After each failure, the planner incrementally switches to different candidates

Figure 8 shows two failure cases of the repeated graph building and the recursive searching. In the upper row of these figures, the system selects the Object *A* as the base and tries the assembly sequence shown in the left. It is the first element of the candidate assembly sequence $\mathbf {C}$. The system builds the regrasp graph and searches the graph to do motion planning in (1–6). In details, the system checks the accessible grasps (the green plots) associated with the initial and goal states, and uses them to build the regrasp graph in (1). Then, the system searches the graph and does motion planning to transfer the Object *A* from the initial state to the goal in (2–4). In (5–6), the system checks the accessible grasps associated with the second object’s initial and goal states. Since there is no accessible grasps (no green plots) associated with the goal state, the system cannot build the third layer of the regrasp graph and cannot find a path. It reports failure and rolls back to use a different assembly sequence.

In the lower row, the system uses the same base but tries a different assembly sequence in $\mathbf {C}$. It builds and searches the graph and performs motion planning in (7–12). Like (1), the system checks the accessible grasps of the base object’s initial and goal states, and builds a graph in (7). Then, it searches the graph, performs motion planning, and successfully finds a way to transfer the base object to the goal. In (11–12), the system checks the grasps of the second object and finds all grasps at the goal are not accessible. It reports failure and rolls back to another assembly sequence again. The searching and rolling back process continues incrementally until a solution is found or all bases and sequences are tried.

**Fig. 9:**
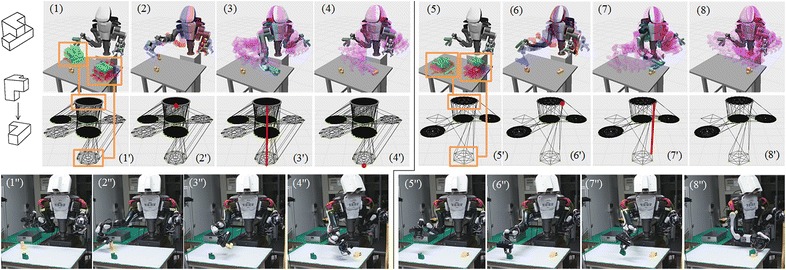
The successful plan. The *third element* in the candidate assembly sequence of the two objects shown in Fig.2 leads to a successful plan. The simulated motion sequence is shown in (1–8). The correspondent regrasp graphs and the paths on the graphs are shown in (1’–8’). (1”–8”) show the correspondent robot execution. The searching process took about 30 s on the Xeon E5-2630 CPU

Figure 9 shows a successful case. Here, the system tries the third assembly order in $\mathbf {C}$. Like the flow in Fig. 8, the system computes the grasps associated with the initial state and goal state of the base object in (1). The accessible grasps are rendered in green. They correspond to the top and bottom layer of the grasp shown in (1’). In (2), the system chooses one candidate from the accessible grasps and does motion planning to pick up the object. The selected grasp corresponds to one node in the top layer of the graph, which is marked with red color in (2’). In (3), the robot picks up Object *A* and transfers it to the goal state using a second motion planning. This corresponds to an edge in (3’) which connects the node in one circle to the node in another. The edge directly connects to the goal without any intermediate placements. After that, the robot moves its arm back at (4), which corresponds to a node in the bottom layer of the graph shown in (4’). In (5), the system computes the grasps associated with the initial and goal states of the second object. The accessible grasps are rendered in green and correspond to the nodes in the top and bottom layer of the regrasp graph shown in (5’). Both initial and goal states have accessible grasps, and it is possible to build the regrasp graph for the second object this time. In (6), the system chooses one feasible grasp and does motion planning to pick up the object. The selected grasp corresponds to the marked node in (6’). In (7), the robot picks up Object *B* and assembles it to its goal state using a second motion planning which corresponds to an edge in (7’). Finally, the robot moves its arm back at (8) and (8’).

The subfigures (2”–4”) and (6”–8”) in the third row show how the robot executes the planned motion. They correspond to (2–4), (6–8) and (2’–4’), (6’–8’) in the first two rows. The whole process is divided at (4/4’/4”) and (5/5’/6”) where (1/1’/2”–4/4’/4”) are picking-up and placing-down the base object and (5/5’/6”–8/8’/8”) are assembling the second object to the base. A video clip that shows both the simulation and real-world execution is available online at: https://www.youtube.com/watch?v=MAT6ucmTRnE.

## Conclusions

This paper presented an integrated assembly and motion planning system which recursively find how to assemble two objects with the help of a horizontal surface as the supporting fixture. The system decides (1) which object is used as the base, (2) how to place the base, and (3) how to assemble the second object to the base. It can find a feasible solution to assemble two objects with completeness. The proposed system is expected to help robot perform assembly tasks automatically, and take the place of the technicians who manually specify the assembly sequences using their experience.

 Currently, the assembly is limited to two objects. Increasing the number of objects would lead to exploded combinatorics and is computationally infeasible. It is therefore a challenging open problem to plan the integrated assembly of more than two objects. In our most recent work [38], we studied a simplified version of this open problem and solved the assembly sequences of 3, 4, and 5 objects with a fixed final assembly pose. Interested readers are recommended to read the paper. Another limitation of the work is that assembly motion is limited to translation. Assembly with rotation is a difficult problem and remains unsolved. The difficulties include planning a specific twist and detecting and taking advantages of contacts. This paper cannot tackle assembly with rotation and is limited to assembly with translational motion. Also, the current system is kinematic. We will add force control to the system in the future and use it to challenge real-world assembly tasks.

## Footnotes

1
http://nextage.kawada.jp/en.

2 The position can be optimized by computing the robot’s manipulability [21, 35].

3 The basic principle is human beings teach the relative poses of the two objects in front of a camera using AR markers. The details can be found in [36]. Practitioners may also use CAD software to pre-define the relative poses.
